# A Cohort Study of Traffic-Related Air Pollution Impacts on Birth Outcomes

**DOI:** 10.1289/ehp.10952

**Published:** 2008-01-23

**Authors:** Michael Brauer, Cornel Lencar, Lillian Tamburic, Mieke Koehoorn, Paul Demers, Catherine Karr

**Affiliations:** 1 School of Environmental Health; 2 Centre for Health Services and Policy Research; 3 Department of Health Care and Epidemiology, The University of British Columbia, Vancouver, British Columbia, Canada; 4 Department of Pediatrics, University of Washington, Seattle, Washington, USA

**Keywords:** air pollution, birth weight, carbon black, carbon monoxide, nitrogen dioxide, nitric oxide, particulate matter, pregnancy, pregnancy outcome, preterm birth, soot, sulfur dioxide, vehicle emissions

## Abstract

**Background:**

Evidence suggests that air pollution exposure adversely affects pregnancy outcomes. Few studies have examined individual-level intraurban exposure contrasts.

**Objectives:**

We evaluated the impacts of air pollution on small for gestational age (SGA) birth weight, low full-term birth weight (LBW), and preterm birth using spatiotemporal exposure metrics.

**Methods:**

With linked administrative data, we identified 70,249 singleton births (1999–2002) with complete covariate data (sex, ethnicity, parity, birth month and year, income, education) and maternal residential history in Vancouver, British Columbia, Canada. We estimated residential exposures by month of pregnancy using nearest and inverse-distance weighting (IDW) of study area monitors [carbon monoxide, nitrogen dioxide, nitric oxide, ozone, sulfur dioxide, and particulate matter < 2.5 (PM_2.5_) or < 10 (PM_10_) μm in aerodynamic diameter], temporally adjusted land use regression (LUR) models (NO, NO_2_, PM_2.5_, black carbon), and proximity to major roads. Using logistic regression, we estimated the risk of mean (entire pregnancy, first and last month of pregnancy, first and last 3 months) air pollution concentrations on SGA (< 10th percentile), term LBW (< 2,500 g), and preterm birth.

**Results:**

Residence within 50 m of highways was associated with a 26% increase in SGA [95% confidence interval (CI), 1.07–1.49] and an 11% (95% CI, 1.01–1.23) increase in LBW. Exposure to all air pollutants except O_3_ was associated with SGA, with similar odds ratios (ORs) for LUR and monitoring estimates (e.g., LUR: OR = 1.02; 95% CI, 1.00–1.04; IDW: OR = 1.05; 95% CI, 1.03–1.08 per 10-μg/m^3^ increase in NO). For preterm births, associations were observed with PM_2.5_ for births < 37 weeks gestation (and for other pollutants at < 30 weeks). No consistent patterns suggested exposure windows of greater relevance.

**Conclusion:**

Associations between traffic-related air pollution and birth outcomes were observed in a population-based cohort with relatively low ambient air pollution exposure.

Numerous studies have indicated associations between exposure to ambient air pollution and adverse pregnancy outcomes. Such associations, if determined to be causal, are likely to result in significant public health impacts given the widespread exposure to air pollution and the fact that low birth weight (LBW) or preterm births are subsequently associated with long-term sequelae such as developmental disability and chronic lung disease (Cano et al. 2001; Dik et al. 2004). Determination of a causal relationship between air pollution and adverse pregnancy outcomes would have implications for burden of disease measures and add to the importance of strategies to mitigate the health effects of air pollution exposure.

Previous studies have been reviewed in detail. Šrám et al. (2005) concluded that evidence is sufficient to support a causal association between ambient concentrations of particulate matter and LBW, but evidence of effects for other pollutants and for other outcomes such as preterm birth is less robust. Maisonet et al. (2004) concluded that studies to date support small effects of air pollution on preterm birth and small for gestational age birth (SGA), but not full-term LBW. In a systematic review, Glinianaia et al. (2004) suggested that evidence of associations with air pollution and fetal growth or pregnancy duration is limited and inconclusive and argued for population-based cohort designs using high-quality individual exposure estimates. These reviews highlight the difficulties in interpreting an evidence base with differences among methods and with important limitations. First, most studies are either time-series studies (Dugandzic et al. 2006; Liu et al. 2003, 2007; Mannes et al. 2005; Sagiv et al. 2005) that relate relatively short-term changes in air pollution concentrations to temporal changes in rates of adverse pregnancy outcomes or, less frequently, cohort analyses that compare outcomes between locations with differing levels of ambient air pollution (Salam et al. 2005) based on interpolated ambient monitoring network data. Between-city comparisons are subject to potential confounding because covariates may be highly correlated with air pollution, whereas time-series studies are problematic to interpret because they relate short-term changes in air pollution that are driven primarily by meteorology to outcomes. They inherently assume that the impact of air pollution on birth outcomes is acute, require knowledge of the relevant periods of pregnancy during which air pollution may have impacts, and are subject to potential confounding by seasonally varying factors. As reviewed by Glinianaia et al. (2004), a number of studies have suggested stronger relationships between birth outcomes and exposure during specific periods of pregnancy based on comparison of statistical effect sizes. However, results across studies have not consistently identified specific periods of exposure that are most closely linked to adverse pregnancy outcomes.

Increasingly, air pollution researchers have identified important spatial variability in air pollution concentrations within airsheds (Hoek et al. 2002b; Lewne et al. 2004; Zhang et al. 2004; Zhu et al. 2004). In many situations these contrasts are of greater magnitude than between-city or temporal contrasts (Jerrett et al. 2005). Such spatial contrasts, primarily related to measures of proximity to traffic corridors, have been associated with a number of health impacts including mortality (Hoek et al. 2002a; Maynard et al. 2007; Miller et al. 2007; Nafstad et al. 2004; Roemer and van Wijnen 2001), asthma and respiratory symptoms (Bayer-Oglesby et al. 2006; Brauer et al. 2002, 2007; Gauderman et al. 2005, 2007; McConnell et al. 2006; Ryan et al. 2005; Smargiassi et al. 2006), and otitis media (Brauer et al. 2006).

Application of within-airshed spatial contrasts in birth outcome studies are few (Leem et al. 2006; Parker et al. 2005; Ritz and Yu 1999; Ritz et al. 2000; Slama et al. 2007; Wilhelm and Ritz 2003, 2005). These studies, though provocative, have been limited largely to Southern California—a metropolitan area with relatively high levels of ambient air pollution. They relied on interpolated ambient monitoring data or simple road proximity measures rather than high-resolution spatial contrasts in concentrations. We sought to assess the relationship between reproductive outcomes and spatial and temporally varying levels of air pollution in the metropolitan area of Vancouver, British Columbia, Canada, a city with relatively low levels of ambient air pollution. We estimated exposures at the individual level, for a population-based cohort using both monitor-based methods and land use regression models based on proximity to traffic sources, land use, population density, and topographic features. Even in Vancouver, an area with a dense ambient monitoring network, exposure assessment based on regulatory monitoring network data is more suited to characterizing temporal variability. Land use regression models, even those with temporal components, as in this analysis, focus on high-resolution spatial variability in air pollutant concentrations.

The literature describing associations between air pollution and birth outcomes has focused on clinically defined outcomes of LBW and preterm birth, defined in a variety of ways, which complicates comparisons. The underlying biological processes—fetal growth restriction and inadequate gestational length—are incompletely understood and imperfectly represented in routinely available perinatal measurements available in vital statistic records. We elected to focus on SGA births as a primary outcome measure, because birth weight as a function of gestational age has a direct effect on perinatal morbidity and mortality (Pollack and Divon 1992).

LBW may result from complex and multiple pathways of fetal growth restriction attributed to maternal, fetal, or placental factors. Three broad categories of biological factors have been suggested to play a role in inadequate fetal gestation: abnormality of the biological clock, abnormal implantation, and infection and inflammation (Mattison et al. 2003).

The current theories provide multiple sites at which environmental factors may influence biological factors to modulate fetal growth and induce preterm birth. However, specific toxicologic mechanisms including relevant timing during gestational development are not known. We explored each of these processes, fetal growth restriction and inadequate gestational length, separately, and explored the influences of exposure timing in early and late pregnancy.

## Methods

### Cohort

The study area is the greater Vancouver metropolitan region. We constructed the cohort by extracting data from a series of linked administrative data sets obtained from the British Columbia Ministry of Health, the British Columbia Vital Statistics Agency, and the British Columbia Perinatal Database Registry. Health data are available through an approved process (Chamberlayne et al. 1998) via the British Columbia Linked Health Database for research purposes and governed by a data access agreement between the researchers and the data stewards. Medical services and hospitalization data were provided and governed by the Ministry of Health, Government of British Columbia; and vital statistics data by the British Columbia Vital Statistics Agency. These data were further merged through an additional data access agreement with records in the provincial perinatal database governed by the British Columbia Reproductive Care Program. The study protocol was approved by the Institutional Review Board (Behavioural Research Ethics Board) of The University of British Columbia. Briefly, we identified vital statistics records for 92,158 children born in the study area during a 4-year period (1999–2002); 77,342 had mothers with verified complete residential history within the study area during the 9 months of pregnancy. Exclusions were made for 976 multiple births, 8 children with no recorded birth weight or parity status, 2,998 with missing maternal age, 1,691 with a missing native status (ethnicity) indicator, and 1,420 who were missing specific census covariates (neighborhood income, maternal education) of interest. From the 77,342 with residential history in the study area, these exclusions left 70,249 subjects (90.8%) for analyses. Additional subjects were excluded from specific (monitoring network-based) analyses if measurements were not available for suitably proximal monitors.

#### Residential history

We compiled maternal residential histories from the beginning of pregnancy until birth from postal codes and associated dates recorded in provincial health plan registry files, and from all hospital discharge and physician billing records for each mother. Approximately 10% of the compiled records included invalid or nonresidential (including urban post office boxes) postal codes and were excluded from the residential history assignment. For each subject a longitudinal residential history was constructed from the remaining data. Where changes in postal codes were observed during the follow-up period, transitions were set as the midpoint between dates if nonoverlapping, or at the first date of the next postal code if overlapping.

### Administrative data sources

#### Birth weight and duration of gestation

We used vital statistics records to identify SGA births, defined as those with birth weights below the 10th percentile of the cohort, stratified by sex, for each week of gestation. LBW at term were those with at least 37 weeks gestation and birth weight < 2,500 g, and preterm births were those with < 37 weeks duration of gestation, as indicated on the vital statistics birth records. Subgroup analyses were conducted for births of < 30, 30–34, and 35–37 weeks gestation and for birth weights below the 5th percentile of the cohort for each week of gestation.

#### Covariates

For each mother we also collected available data for several covariates of interest that may be associated with the health outcomes. Sex, parity, and the month and year of birth were available from Vital Statistics records. First Nations (“status Indians”) status of the baby was obtained for each individual from hospital discharge records. Maternal age and maternal smoking during pregnancy was obtained from Perinatal Database Registry files that were linked to each individual birth record. Because no individual-level data were available for income and maternal level of education, we assigned subjects to neighborhood-level income quintiles and maternal education quartiles using Census data based on their residence at the resolution of the census dissemination area (DA). Dissemination areas are the smallest geographic areas for which all Canadian Census data are disseminated and corresponds to one or more neighboring blocks with target populations of 400–700 persons (Puderer 2001).

### Air pollution

#### Monitoring network data

Exposure to air pollution for each cohort member was assigned by three different approaches, two based on the regulatory monitoring network and one based on dedicated sampling campaigns. The regulatory monitoring network was operated by the British Columbia Ministry of Environment and Metro Vancouver and includes daily measurements at 24 monitors for ozone, 22 for nitric oxide/ nitrogen dioxide, 14 for sulfur dioxide, 19 for carbon monoxide, 19 for particulate matter < 10 μm in aerodynamic diameter (PM_10_), and 7 for PM < 2.5 μm in aerodynamic diameter (PM_2.5_). The monitoring data were assigned to individuals at their 6-digit postal code of residence. The 6-digit postal code typically corresponds to one block-face in urban areas; areas may be considerably larger in rural areas with low population density.

Concentrations were assigned to postal codes by nearest monitor and inverse-distance weighting (IDW) approaches. This approach provided high temporal resolution (daily measures for most days) with less precise spatial resolution than land use regression estimates. For the nearest monitor assignment, we assigned for each day a concentration from the operational monitor closest to the postal code of interest and within 10 km. We then computed monthly averages for each individual for the full duration of their pregnancy. For the IDW approach we used an inverse-distance (1/distance) weighted average of the three closest monitors within 50 km to compute a monthly mean concentration. For both approaches, a month was considered missing if there was a gap of > 5 consecutive days in air monitoring data or if there were > 10 missing days in a given month. Using the monthly averages, we then computed mean exposures for each mother for the full duration of pregnancy, the first and last 30 days of pregnancy, and the first and last 3 months of pregnancy. Exposures were updated with change in postal code of residence and weighted by time spent in multiple residences. Postal code information for mothers was obtained from the provincial health registration and health care contact records.

#### Land use regression model

Exposure assessment based on a land use regression (LUR) model developed for the study region (Henderson et al. 2007) provided improved local spatial resolution. Briefly, 116 passive samplers to collect NO and NO_2_ were deployed for two 14-day periods at 116 sites in the study area. Mean concentrations during these two periods were highly correlated with and closely approximated annual averages from regulatory monitoring network data. In addition, PM_2.5_ mass was measured once at a subset of 25 locations during a 2-month sampling period. Integrated 1-week average PM_2.5_ samples were collected on Teflon (Teflo; Pall Corp., East Hills, NY, USA) filters using Harvard Impactors (Air Diagnostics and Engineering, Harrison, ME, USA) at a flow rate of 4 L/min. Five sampling units were rotated between the 25 sites and one was collocated with a tapered element oscillating microbalance (TEOM; Thermo Electron Corp., East Greenbush, NY, USA) sampler at a regulatory monitoring network station. For a subset of 39 sites, we measured short-term levels of particle absorbance (black carbon) on one occasion using a particle soot absorption photometer (Radiance Research, Seattle, WA, USA) in a mobile monitoring platform. These measurements were adjusted for temporal variation based on repeated measurements at a centrally located site to result in estimated annual average concentrations. We have previously demonstrated strong correlations (*R*^2^ 0.7–0.8) (Noullett et al. 2006; Rich 2003) between particle absorbance and traditional measurements of elemental carbon (Cyrys et al. 2003).

For each of the 116 (and the subsets of 25 and 39) measurement sites, 55 variables were generated in a geographic information system (GIS) (ArcGIS; ESRI, Redlands, CA, USA), and linear regression models of NO, NO_2_ and black carbon were built with the most predictive covariates. For NO, the model had an *R*^2^ of 0.62 and included the number of major roads within 100-m and 1,000-m radius circular buffers of the measurement sites, the number of secondary roads within a 100-m buffer, the population density within a 2,500-m radius, and elevation. For NO_2_, the model (*R* ^2^ = 0.56) included the same variables as well as the amount of commercial land use within 750 m. For PM_2.5_ the model (*R*^2^ = 0.52) included the amount of commercial and industrial land use within 300 m, the amount of residential land use within 750 m, and elevation. For black carbon the model (*R*^2^ = 0.56) included the number of secondary roads within a 100-m buffer, distance to the nearest highway, and the amount of industrial land use within 750 m. Evaluations, based on comparison to additional measurements and cross-validation analysis, indicated that the PM_2.5_ and black carbon models performed much more poorly than did the NO or NO_2_ models. Using the LUR models, smooth spatial surfaces of predicted (annual average) concentrations were generated for the entire study area at a resolution of 10 m. The surfaces were then smoothed (Focal Statistics, ArcGis Spatial Analyst; ArcGIS) to remove abrupt changes and edge effects so as to more accurately reflect the measured effect of proximity to roadways (Gilbert et al. 2003). For each LUR model, the corresponding monitoring network data for each pollutant were fit with a monthly dummy variables and a covariate for linear trend (Times Series Forecasting System, version 9; SAS Institute Inc., Cary, NC, USA). For black carbon, the PM_2.5_ trend was used because there were no corresponding regulatory monitoring network data. From these models, we applied month–year adjustment factors to each surface to estimate monthly average concentrations. Using these, we then computed individual subject exposures for the same exposure windows as described above for the monitor-based approaches.

#### Road proximity

Finally, we calculated road proximity for home postal codes of all cohort members. Road classifications (DMTI ArcView street file data set for British Columbia, Canmap Streetfiles, version 2006.3; DMTI Spatial, Markham, Ontario, Canada) were used to determine whether a home postal code was within 50 or 150 m of an expressway or primary highway, within 50 or 150 m of a secondary highway or major road/arterial road, or within 150 m of a secondary highway or major road or within 50 m of an expressway or primary highway.

## Results

Of the 70,249 live births considered in analysis, 36,138 (51.4%) were male and 542 (0.77%) had First Nations status indicated. Mean parity was 1.8 and the mean maternal age was 31.1 years. A total of 7.4% (5,198) of the cohort reported maternal smoking during pregnancy. The mobility of the cohort as ascertained from residential histories indicated that 60.7% (42,649) had a single address during pregnancy, whereas 34.9% (24,537) had two addresses, and 4.4% (3,063) had three to five addresses. 89.2% of the postal codes were referenced to a block face, and an additional 8.5% referenced to a block.

As expected for the greater Vancouver area, air pollution concentrations were low relative to air quality standards and international guidelines (Table 1). Mean concentrations and ranges at the middle of the concentration distributions were similar for the monitor-based and the LUR estimates. As expected, the LUR estimates had smaller minimum and greater maximum values than the IDW estimates.

Correlations between IDW estimates of CO, NO, NO_2_, and SO_2_ were all > 0.8 and negatively correlated with O_3_ (*r* = –0.7 to –0.8). Correlations between LUR estimates CO, NO, NO_2_, SO_2_ and PM_10_ or PM_2.5_ were lower (*r* = 0.1 to 0.5). Correlations for the same pollutant between IDW and LUR estimates were moderate (*r* = 0.55 for NO, 0.37 for NO_2_) except for the LUR PM_2.5_ estimates, which were not correlated with IDW estimates of either PM_2.5_ or PM_10_. IDW and nearest monitor estimates for the same pollutant were all highly correlated (*r* = 0.70 to 0.89). Given these high correlations, and because the nearest monitor exposure estimates displayed sharp spatial patterns in exposure related to monitor location (Marshall et al. 2008), we focused on the IDW estimates.

Exposures estimated for various periods of pregnancy were, in general, highly correlated with the full term of pregnancy average exposure, limiting our ability to assess the impact of exposure during specific windows of pregnancy. Correlations between exposure during the first or last 3 months of pregnancy and the full term of pregnancy were 0.63–0.89 for IDW estimates of gaseous pollutants, 0.48–0.64 for IDW estimates of PM, and 0.59–0.94 for LUR estimates.

Table 2 presents the prevalence of SGA, LBW at term, and preterm births in the cohort. As reported by others (Ghosh et al. 2007), we observed higher rates of SGA for females (104.9 vs. 103.6 per 1,000 live births) and higher rates of preterm birth for males (56.9 vs. 49.6 per 1,000 live births).

For NO, NO_2_, CO, PM_10_, PM_2.5_, and black carbon we observed small but consistent increased risks of SGA (Table 3). Odds ratios (ORs) for exposure estimates based on the nearest monitor approach were similar to those for the inverse distance weighted estimates [e.g., for NO, OR = 1.03; 95% confidence interval (CI), 1.01–1.05 for nearest, vs. OR = 1.05; 95% CI, 1.03–1.08 for IDW] and are therefore not presented further in the analysis. Because O_3_ was highly negatively correlated with all of the primary traffic-related air pollutants (*r* = –0.83 for IDW CO; *r* = –0.86 for IDW NO), associations were largely protective (e.g., OR = 0.89; 95% CI, 0.84–0.94 for IDW SGA) and are not presented further.

Although our prior expectations were that LUR exposure assessments would provide increased precision and variability of individual exposure, associations were not consistently larger for the LUR estimates, although their CIs were smaller in most cases. Similar results (not shown) were found for the subset of SGA births with birth weights below the 5th percentile of the cohort for each week of gestation. We did not observe (results not shown) any consistent patterns indicating larger effect estimates for exposures during specific periods of pregnancy. ORs for early (first month, first 3 months) or late pregnancy (last month, last 3 months) exposure windows were remarkably similar to those for the full duration of pregnancy. Similar patterns were observed for LBW (Table 4), although associations, except for IDW NO_2_, did not reach statistical significance because of the smaller number of LBW cases.

Tables 5 and 6 present the analysis of the simple road proximity measures. In contrast to the LUR models that indicated a small magnitude increased risk of SGA and LBW in relation to traffic-related air pollutants, a strong association was observed for mothers who resided within 50 m of an expressway or highway. Although the number of subjects meeting this criterion was small (0.25% of all births for SGA), there was a 26% increased risk of an SGA birth compared with those mothers residing > 50 m from an expressway or highway (mean of > 21,000 vehicles per day). For LBW, we observed an 11% increase in risk for this group. No increased risk was observed for those living within 150 m of a highway or within 50 m of a major road (mean of 15,000–18,000 vehicles per day). The LUR models are derived in part from the density of roads of within specific radii, but also include land use and population density variables. Simple road proximity measures were not strongly predictive of measured concentrations in the LUR models (Henderson et al. 2007).

For the preterm birth outcome of < 37 weeks, we did not observe any consistent associations with any of the pregnancy average exposure metrics except for PM_2.5_ (IDW: OR = 1.06; 95% CI, 1.01–1.11). In addition, pregnancy average PM_2.5_ exposure was related to preterm births < 35 (IDW: OR = 1.12; 95% CI, 1.02–1.24) and < 30 weeks (IDW: OR = 1.13; 95% CI, 0.92–1.39). For the outcome of birth < 30 weeks, although there were very few cases, we found associations with the different pregnancy average exposure metrics that were similar to those observed for SGA (Table 7). No associations were observed between the simple road proximity measures and preterm birth < 37 weeks, and there were no cases of births < 30 weeks that were within 50 m of a highway. In addition, there were no consistent trends for early or late gestational period exposure to be more strongly associated with preterm births.

## Discussion

We explored several outstanding questions in air pollution reproductive epidemiology in a population-based study using individual estimates of exposure to air pollutants. We observed small-magnitude associations between a number of traffic-related pollutants and SGA birth weight. Consistent associations were also observed between PM_2.5_ and preterm births (< 37 weeks). For other pollutants, associations with gestational duration were observed only for the small subset of births at < 30 weeks gestation. Given high correlations between NO, NO_2_, CO, and SO_2_, it was not possible to differentiate impacts of specific pollutants. Associations with SGA were, however, generally of lower magnitude for PM_10_ and PM_2.5_, and we did not detect any associations with black carbon, although our black carbon model performed relatively poorly in evaluation comparisons and was limited by a small number of measurements and seasonal adjustment by PM_2.5_ rather than black carbon trends. Our results suggest a general association between pollutants dominated by traffic sources (NO_x_ and CO) and LBW in this study. This conclusion is supported by our finding of a strong association between residence within 50 m of a highway or expressway, but not other measures of traffic proximity, and both SGA and LBW at full term.

Overall, results from the traffic-based land use regression models agree with analyses based on exposures estimated from the ambient monitoring network. Although effect estimates based on LUR models did have somewhat smaller CIs than those based on monitoring network data, they did not indicate increased estimates of effect, as one might expect given improved spatial resolution of the LUR models and the potential for reduced exposure misclassification. It is possible that LUR exposure estimates may be more appropriate for primary pollutants, such as NO and black carbon, that vary the most spatially, whereas monitor-based estimates are more appropriate for secondary pollutants such as NO_2_ and PM_2.5_ that display less spatial heterogeneity. In contrast to the improved spatial resolution of land use regression-based exposure estimates, exposures determined from ambient monitors are directly related to a larger number of measurements, include more precise temporal information, and capture a different spatial scale of variability in ambient air pollution (Marshall et al. 2008). Exposure estimates from land use regression and monitoring network data for the same pollutant were only moderately correlated and appeared to be somewhat independent, with each capturing different aspects of spatiotemporal variability in exposure (Marshall et al. 2008).

Because exposures were estimated only for home addresses, it is also possible that subject mobility was related to varying degrees of exposure misclassification for the different modeling approaches. An evaluation of the LUR and IDW models for (short-term) measured personal exposures of pregnant women indicated that for NO and NO_2_, especially for those who were the least mobile, LUR models were a stronger predictor of personal exposure and better explained between-subject variability in exposure. Monitor-based estimates better explained within-subject (temporal) variability in exposure. For PM_2.5_ and black carbon, monitor-based estimates (of PM_2.5_) were more highly correlated with personal exposures than were the LUR models (Nethery et al. 2007),

Overall, these differences between the monitor-based and LUR model exposure estimates suggest that our finding of risks associated with both types of exposure estimates indicate some independence of these risks. In analysis of air pollution and childhood lung function, independent effects from both regional and local traffic-related air pollution have been reported (Gauderman et al, 2007).

Although simple measures of road proximity have been shown to predict substantially smaller percentages of variability in measured concentrations of NO_x_, PM_2.5_, and black carbon compared with LUR models (Brauer et al. 2003; Henderson et al. 2007), road distance measures are straightforward, precise, directly relevant to land use policy, and easy to assess and apply in areas without high monitor density. In particular, it is possible that the LUR model estimates, though more specific measures of individual air pollutants, may incorporate more exposure mis-classification to the harmful components of traffic than do the measures of road proximity. We found increased risks of SGA and LBW only for those subjects closest to roads of highest traffic intensity in our study area.

Our findings of associations between air pollution and SGA are consistent with reports from a number of studies (Dejmek et al. 1999; Hansen et al. 2007; Liu et al. 2003, 2007; Parker et al. 2005) and conclusions of reviews (Maisonet et al. 2004; Šrám et al. 2005), including studies that focus on spatial contrasts in exposure. For example, studies in Southern California (Ritz and Yu 1999; Wilhelm and Ritz 2005), which has a relatively dense ambient monitoring network, found that exposure to higher levels of ambient CO, based on the nearest monitoring stations within 2 miles of the mother’s home address, during the last trimester was associated with increased risk of LBW (OR = 1.22; 95% CI, 1.03–1.44 for 3-month average exposures > 5.5 ppm). Similar effects were also observed for PM_10_, with stronger associations found for subjects living within 1 mile of a monitoring station. Most recently, Slama and colleagues (2007) applied LUR models for PM_2.5_, NO_2_, and black carbon to a smaller cohort of 1,016 full-term births (≥ 37 weeks) > 2,500 g in Munich. An increased prevalence ratio of 1.13 (95% CI, 1.00–1.29) for birth weight < 3,000 g at term was associated with a 1-μg/m^3^ increase in PM_2.5_ concentration, with associations also observed for increased concentrations of black carbon or NO_2_.

For preterm births, our study, even with its large population, had limited numbers of cases with which to detect a relationship with air pollution. We found consistent associations with PM_2.5_ but not other pollutants for births < 37, 35, or 30 weeks. For risk of very preterm birth (< 30 weeks), we observed elevated ORs for a larger number of pollutants (NO, NO_2_, CO, PM_10_, and PM_2.5_ but not SO_2_). As with SGA, we did not observe any association with black carbon. In addition to the poor performance of our black carbon LUR model in evaluation tests, the application of this model was further limited by our need to use PM_2.5_ monitoring data, as opposed to black carbon measurements, for seasonal adjustment.

A general limitation for our modeling approach for analysis of preterm births arises from the fact that entire pregnancy exposure averaging periods will, by definition, be different for cases and noncases. Further, the last 3 months and last month of pregnancy occur at different gestational periods for cases and non-cases. The importance of these differences in exposure is less significant in our data, because air pollution exposures for different pregnancy periods were rather highly correlated.

Measures of road proximity, which do not suffer from this potential problem, were not associated with preterm births. This analysis, however, was limited by the low numbers of mothers with preterm births who resided < 50 m of a highway (for example, no births < 30 weeks).

In this analysis we did not identify specific exposure windows (early or late pregnancy) of greater or lesser relevance for SGA or preterm birth, although exposures from different periods of pregnancy were highly correlated. Reports from other studies have been inconsistent with regard to the importance of early or later pregnancy exposures. The recent study of Slama and colleagues (2007) showed a tendency for third-trimester exposures to exhibit stronger associations with LBW, but these associations were also highly correlated with exposures averaged over the full pregnancy. Multiple potential mechanisms by which air pollution may affect fetal growth and birth weight have been proposed, but there is little information regarding specific etiology on which greater importance of specific exposure windows can be based.

This study had several advantages over previous analyses of the relationship between air pollution and birth outcomes. First, the study was population based and estimated exposures at the individual level using both interpolated ambient monitoring network data and temporally adjusted LUR models to characterize spatiotemporal variability in exposure to a greater degree than in previous studies. A recent exception is the study from Munich, which also applied temporally adjusted LUR models, although to a smaller and more restrictive cohort (Slama et al. 2007). In addition, we accounted for residential mobility during pregnancy when assigning exposures. Failure to account for mobility, especially in studies such as ours using very small areas to assign exposures, is likely to result in misclassified exposures (Fell et al. 2004). In our study we identified 35% of the population as changing a residence during pregnancy, highlighting the importance of accounting for mobility in residence-based assessment of exposure. Mobility rates among pregnant women reported in the literature range from 12% (Fell et al. 2004) to 33% (Canfield et al. 2006), although most studies were small population case–control studies. Further, by a unique linkage of multiple databases, we acquired individual information on several important covariates.

Largely because of the use of administrative databases, this study does have several important limitations. We defined fetal growth restriction as a weight below the 10th percentile for gestational age. This definition is imperfect and controversial because it does not make a distinction among fetuses who are constitutionally small, growth restricted and small, and growth restricted but not small. The data elements in our administrative data set did not allow us to make these distinctions (e.g., we did not have data on parental size or birth length). The definition used, however, represents a common definition of fetal growth restriction and increases the opportunity for comparisons among studies.

We were also limited by unavailability of individual data for indicators of socioeconomic status. In this case we had to rely on neighborhood-level census data, where there is a likelihood of misclassification at the individual level. In addition, no information was available on maternal ethnicity, except First Nations status, as ethnicity is known to affect birth weight distributions (Janssen et al. 2007). We also had no information regarding nutrition or prenatal care. Further, although unlike most previous studies we did account for residential mobility in assigning exposures, our ascertainment of mobility was imperfect because we had no information on the actual moving date; moving was assumed from consistent changes in address information collected during health care system contacts. This limitation may have affected our sensitivity to detect more acute effects of air pollution—for example, impacts on preterm birth. In addition, exposures were based only on residential address and did not consider time weighting by other important microenvironments, which more closely approximates measured exposures (Nethery et al. 2007).

## Conclusions

Our findings in a population-based study add to an expanding literature that links several traffic-derived air pollutants (e.g., NO, NO_2_, CO) to adverse birth outcomes, particularly increased risk of SGA birth weight. In addition, we observed consistent associations between PM_2.5_ exposure and risk of preterm birth. The overall importance of proximity to highways, even in an area with relatively low levels of ambient air pollutants, suggests opportunities for urban planning approaches to prevention. The toxicologically relevant components of traffic-related air pollution remain unclear and deserve increased attention in future studies.

## Figures and Tables

**Table 1:** Distributions of air pollution concentrations estimated by different approaches.

Pollutant	Model	Mean	Min	Max	IQR
NO	Nearest	23.7	0.5	93.3	14.3
NO	IDW	22.1	2.9	63.6	14.9
NO	LUR	30.7	1.4	145.5	14.4
NO_2_	Nearest	34.4	9.7	63.8	9.3
NO_2_	IDW	32.5	15.3	53.6	11.3
NO_2_	LUR	31.6	0.0	63.8	9.3
CO	Nearest	633.1	124.3	1,409.0	194.7
CO	IDW	613.8	305.4	1,062.3	172.1
PM_2.5_	Nearest	5.3	0.3	37.0	1.0
PM_2.5_	IDW	5.1	1.0	7.6	1.1
PM_2.5_	LUR	4.0	0.0	11.3	1.5
BC	LUR	1.6	0.0	6.2	1.2
PM_10_	Nearest	12.7	5.6	35.4	1.6
PM_10_	IDW	12.5	8.4	16.6	1.4
SO_2_	Nearest	5.7	0.0	24.9	3.5
SO_2_	IDW	5.3	0.3	17.8	3.0
O_3_	Nearest	28.0	2.3	69.2	8.2
O_3_	IDW	28.3	10.4	48.2	8.4

Abbreviations: BC, black carbon; IQR, interquartile range; Max, maximum; Min, minimum. Weighted concentrations of 3 monitors within 50 km of residential postal code. Nearest: concentration from nearest monitor within 10 km of residential postal code. All concentrations were computed for full duration of pregnancy. All concentrations in μg/m^3^ except for BC, which is in 10^–5^ particles/m.

**Table 2:** Prevalence of SGA, LBW, and preterm birth in the cohort (*n* = 70,249).

Outcome	No. (%)
SGA (< 10th percentile)	6,939 (10.4)
LBW (< 2,500 g at ≥ 37 weeks^a^)	894 (1.3)
Gestation < 37 weeks	3,748 (5.3)
Gestation < 35 weeks	820 (1.2)
Gestation < 30 weeks	170 (0.2)

a *n* = 66,501 births at full (≥ 37 weeks) term.

**Table 3:** Crude and adjusted ORs^a^ for SGA.

Exposure (entire pregnancy)	SGA cases (*n*)	Crude OR (95% CI)	Adjusted^b^ OR (95% CI)
NO–IDW (10 μg/m^3^)	6,351	1.03 (1.00–1.05)	1.05 (1.03–1.08)
NO–LUR (10 μg/m^3^)	6,750	1.02 (1.01–1.04)	1.02 (1.00–1.04)
NO_2_–IDW (10 μg/m^3^)	6,351	1.12 (1.08–1.16)	1.14 (1.09–1.18)
NO_2_–LUR (10 μg/m^3^)	6,750	1.02 (0.99–1.05)	0.99 (0.96–1.02)
CO–IDW (100 μg/m^3^)	6,351	1.03 (1.01–1.05)	1.06 (1.03–1.08)
PM_2.5_–IDW (1 μg/m^3^)	6,307	0.99 (0.97–1.01)	1.02 (0.98–1.05)
PM_2.5_–LUR (1μg/m^3^)	6,750	1.03 (1.02–1.05)	1.02 (1.00–1.03)
BC–LUR (10^–5^/m)	6,750	1.03 (1.01–1.05)	1.01 (0.99–1.03)
PM_10_–IDW (1 μg/m^3^)	6,351	1.02 (1.00–1.05)	1.02 (0.99–1.05)
SO_2_–IDW (1 μg/m^3^)	6,351	1.01 (1.00–1.02)	1.01 (1.00–1.02)

BC, black carbon.

a ORs per standardized increases (indicated in parentheses), roughly corresponding to interquartile range.

b Adjusted for infant sex, First Nations status, parity, maternal age, maternal smoking during pregnancy, month–year of birth, income (quintile-census), maternal education (quartile-census).

**Table 4:** Crude and adjusted ORs^a^ for LBW at full (≥ 37 weeks) term.

Exposure (entire pregnancy)	LBW cases (*n*)	Crude OR (95% CI)	Adjusted^b^ OR (95% CI)
NO–IDW (10 μg/m^3^)	894	1.02 (0.96–1.09)	1.03 (0.96–1.10)
NO–LUR (10 μg/m^3^)	874	1.02 (0.98–1.08)	1.01 (0.96–1.07)
NO_2_–IDW (10 μg/m^3^)	894	1.12 (1.02–1.23)	1.11 (1.01–1.23)
NO_2_–LUR (10 μg/m^3^)	874	1.01 (0.94–1.09)	0.97 (0.89–1.05)
CO–IDW (100 μg/m^3^)	894	1.02 (0.96–1.07)	1.02 (0.96–1.09)
PM_2.5_–IDW (1 μg/m^3^)	889	0.98 (0.92–1.06)	0.98 (0.92–1.05)
PM_2.5_–LUR (1μg/m^3^)	874	1.05 (1.02–1.09)	1.03 (0.99–1.07)
BC–LUR (10^–5^/m)	874	1.03 (0.97–1.09)	1.00 (0.95–1.07)
PM_10_–IDW (1 μg/m^3^)	894	1.01 (0.95–1.08)	1.01 (0.95–1.08)
SO_2_–IDW (1 μg/m^3^)	894	1.00 (0.97–1.02)	0.99 (0.97–1.02)

BC, black carbon.

a ORs per standardized increases (indicated in parentheses), roughly corresponding to interquartile range.

b Adjusted for infant sex, First Nations status, parity, maternal age, maternal smoking during pregnancy, month–year of birth, income (quintile-census), maternal education (quartile-census).

**Table 5:** Crude and adjusted ORs for SGA and road proximity.

Measure	No. of SGA cases (% of total subjects)	Crude OR (95% CI)	Adjusted^a^ OR (95% CI)
< 150 m highway^b^ or < 50 m major road^c^	1,218 (1.73)	1.01 (0.95–1.08)	0.99 (0.92–1.06)
< 50 m highway	176 (0.25)	1.31 (1.12–1.54)	1.26 (1.07–1.49)
< 150 m highway	413 (0.59)	0.96 (0.86–1.07)	0.93 (0.83–1.03)
< 50 m major road	842 (1.20)	1.04 (0.97–1.13)	1.03 (0.95–1.12)
< 150 m major road	1,611 (2.29)	1.05 (0.99–1.12)	1.04 (0.98–1.11)

a Adjusted for infant sex, First Nations status, parity, maternal age, maternal smoking during pregnancy, month–year of birth, income (quintile-census), maternal education (quartile-census).

b DMTI type 1 and 2 road (expressway: 114,000 vehicles/day; principal highway: 21,000 vehicles/day).

c DMTI type 3 and 4 road (secondary highway: 18,000 vehicles/day; major road: 15,000 vehicles/day).

**Table 6:** Crude and adjusted odds ratios for LBW at full term and road proximity.

Measure	No. of LBW cases (% of total subjects)	Crude OR (95% CI)	Adjusted^a^ OR (95% CI)
< 150 m highway^b^ or < 50 m major road^c^	358 (0.51)	0.98 (0.82–1.17)	0.95 (0.79–1.13)
< 50 m highway	49 (0.07)	1.32 (0.87–2.00)	1.22 (0.81–1.87)
< 150 m highway	124 (0.18)	1.06 (0.80–1.40)	1.01 (0.76–1.33)
< 50 m major road	250 (0.36)	0.97 (0.79–1.20)	0.96 (0.77–1.18)
< 150 m major road	476 (0.68)	0.95 (0.81–1.12)	0.94 (0.79–1.10)

a Adjusted for infant sex, First Nations status, parity, maternal age, maternal smoking during pregnancy, month–year of birth, income (quintile-census), maternal education (quartile-census).

b DMTI type 1 and 2 road (expressway: 114,000 vehicles/day; principal highway: 21,000 vehicles/day).

c DMTI type 3 and 4 road (secondary highway: 18,000 vehicles/day; major road: 15,000 vehicles/day).

**Table 7:** Crude and adjusted ORs^a^ for preterm births < 30 weeks.

Exposure (entire pregnancy)	Cases (*n*)	Crude OR (95% CI)	Adjusted b OR (95% CI)
NO–IDW (10 μg/m^3^)	170	1.19 (1.04–1.35)	1.26 (1.08–1.47)
NO–LUR (10 μg/m^3^)	166	1.08 (0.97–1.19)	1.05 (0.94–1.18)
NO_2_–IDW (10 μg/m^3^)	170	1.13 (0.92–1.39)	1.12 (0.89–1.40)
NO_2_–LUR (10 μg/m^3^)	166	1.11 (0.95–1.31)	1.08 (0.91–1.29)
CO–IDW (100 μg/m^3^)	170	1.13 (1.01–1.28)	1.16 (1.01–1.33)
PM_2.5_–IDW (1 μg/m^3^)	168	0.99 (0.86–1.14)	1.13 (0.92–1.39)
PM_2.5_–LUR (1μg/m^3^)	166	1.08 (1.00–1.18)	1.07 (0.98–1.16)
BC–LUR (10^–5^/m)	166	1.01 (0.89–1.15)	0.99 (0.87–1.13)
PM_10_–IDW (1 μg/m^3^)	170	1.11 (0.97–1.29)	1.13 (0.95–1.35)
SO_2_–IDW (1 μg/m^3^)	170	1.02 (0.96–1.08)	1.02 (0.96–1.09)

BC, black carbon.

a ORs per standardized increases (in parentheses), roughly corresponding to interquartile range.

b Adjusted for infant sex, First Nations status, parity, maternal age, maternal smoking during pregnancy, month–year of birth, income (quintile-census), maternal education (quartile-census).
